# Normal tissue complication probability model parameter estimation for xerostomia in head and neck cancer patients based on scintigraphy and quality of life assessments

**DOI:** 10.1186/1471-2407-12-567

**Published:** 2012-12-04

**Authors:** Tsair-Fwu Lee, Pei-Ju Chao, Hung-Yu Wang, Hsuan-Chih Hsu, PaoShu Chang, Wen-Cheng Chen

**Affiliations:** 1 Medical Physics and Informatics Laboratory, Department of Electronics Engineering, National Kaohsiung University of Applied Sciences, Kaohsiung, Taiwan, ROC; 2 Department of Radiation Oncology, Chang Gung Memorial Hospital-Kaohsiung Medical Center, Chang Gung University College of Medicine, Kaohsiung, Taiwan, ROC; 3 Department of Radiation Oncology, Kaohsiung Medical University Hospital, Kaohsiung, Taiwan, ROC; 4 Department of Medical Imaging and Radiological Sciences, Kaohsiung Medical University, Kaohsiung, Taiwan, ROC; 5 Departments of Radiation Oncology, Chang Gung Memorial Hospital, Chiayi, Taiwan, ROC

**Keywords:** NTCP, Xerostomia, Scintigraphy, Quality of Life (QoL), Quantitative Analysis of Normal Tissue Effects in the Clinic (QUANTEC)

## Abstract

**Background:**

With advances in modern radiotherapy (RT), many patients with head and neck (HN) cancer can be effectively cured. However, xerostomia is a common complication in patients after RT for HN cancer. The purpose of this study was to use the Lyman–Kutcher–Burman (LKB) model to derive parameters for the normal tissue complication probability (NTCP) for xerostomia based on scintigraphy assessments and quality of life (QoL) questionnaires. We performed validation tests of the Quantitative Analysis of Normal Tissue Effects in the Clinic (QUANTEC) guidelines against prospectively collected QoL and salivary scintigraphic data.

**Methods:**

Thirty-one patients with HN cancer were enrolled. Salivary excretion factors (SEFs) measured by scintigraphy and QoL data from self-reported questionnaires were used for NTCP modeling to describe the incidence of grade 3^+^ xerostomia. The NTCP parameters estimated from the QoL and SEF datasets were compared. Model performance was assessed using Pearson’s chi-squared test, Nagelkerke’s R^2^, the area under the receiver operating characteristic curve, and the Hosmer–Lemeshow test. The negative predictive value (NPV) was checked for the rate of correctly predicting the lack of incidence. Pearson’s chi-squared test was used to test the goodness of fit and association.

**Results:**

Using the LKB NTCP model and assuming *n=*1, the dose for uniform irradiation of the whole or partial volume of the parotid gland that results in 50% probability of a complication (TD_50_) and the slope of the dose–response curve (*m*) were determined from the QoL and SEF datasets, respectively. The NTCP-fitted parameters for local disease were TD_50_=43.6 Gy and *m=*0.18 with the SEF data, and TD_50_=44.1 Gy and *m=*0.11 with the QoL data. The rate of grade 3^+^ xerostomia for treatment plans meeting the QUANTEC guidelines was specifically predicted, with a NPV of 100%, using either the QoL or SEF dataset.

**Conclusions:**

Our study shows the agreement between the NTCP parameter modeling based on SEF and QoL data, which gave a NPV of 100% with each dataset, and the QUANTEC guidelines, thus validating the cut-off values of 20 and 25 Gy. Based on these results, we believe that the QUANTEC 25/20-Gy spared-gland mean-dose guidelines are clinically useful for avoiding xerostomia in the HN cohort.

## Background

Head and neck (HN) cancer is a leading cause of cancer mortality in Taiwan, and radiotherapy (RT) plays an important role in its treatment. Xerostomia is a common complication after RT for HN [1-5]. Severe xerostomia is defined as long-term salivary dysfunction compared with the pre-RT function, based on the Late Effects of Normal Tissues–Subjective, Objective, Management, Analytic (LENT–SOMA) criteria [6-8].

Whole-mouth salivary function has been shown to be related to quality of life (QoL) [9,10] and has been used to compare different treatment strategies in clinical oncology trials. Kakoei et al. [11] have shown that the decrease in saliva and xerostomia resulting from RT can negatively affect QoL for patients who undergo RT. Several prospective studies conducted over the past decade have reported the RT dose constraints to allow preservation of parotid gland function based on salivary flow measurements or salivary gland scintigraphy [10,12-14].

In the present prospective study, we longitudinally observed parotid gland function using salivary scintigraphy to measure the salivary excretion factor (SEF) in patients receiving intensity-modulated radiotherapy (IMRT). Moreover, a self-reported QoL questionnaire (QLQ-C30) and a xerostomia-specific questionnaire (QLQ-H&N35) were completed by patients before RT and periodically after therapy to assess the interrelationships with salivary function. The normal tissue complication probability (NTCP) model proposed by Lyman has been used to determine the dose for uniform irradiation of the whole or partial volume of the parotid gland that results in 50% probability of a complication (TD_50_) in patients with local disease [15,16]. NTCP-fitted parameters for patients with local disease were investigated using both datasets.

## Methods

### Study population

Between August 2007 and June 2008, 65 HN cancer patients who had undergone primary or postoperative RT for various malignancies were initially included in the study. Patients who suffered from Sјögren’s syndrome or any other medical cause of xerostomia were excluded. The use of any medication known to affect salivary gland function was prohibited. After eliminating patients because of missed appointments, refusal, and organizational problems, it was possible to objectively evaluate parotid gland function using scintigraphy and QoL questionnaires after RT initiation in 31 patients. The present prospective study enrolled these 31 HN cancer patients who received primary (n=15) or post-operative RT (n=16) with IMRT at Chiayi Chang Gung Memorial Hospital of the Chang Gung Medical Foundation. Nineteen patients received concurrent chemotherapy: 18 received five to seven courses of weekly cisplatin (40 mg/m^2^ CDDP), and one received two courses of a PF regimen (80 mg/m^2^ CDDP on day 1 + 800 mg/m^2^ 5-FU on days 1–5, every 21 days). Five of these patients received additional adjuvant chemotherapy with a PF regimen for two to three courses (n=4) or a TEF regimen (60 mg/m^2^ taxol on day 1 + 20 mg/m^2^ CDDP on day 1 + 800 mg/m^2^ 5-FU on days 1–2) for one course (n=1).

Patients with successful salivary flow scintigraphy imaging and full completion of QoL questionnaires before and during 1 year after treatment were included. No data were missing for these 31 patients. This study was approved by the institutional review board of the hospital (IRB-95-1430B).

### RT techniques

Patients were immobilized from head to shoulders using a commercially available thermoplastic mask and/or an individually customized bite block. Computed tomography (CT) images (2.5-mm slice thickness) acquired from the top of the vertex to the level of the carina, containing 512 × 512 pixels in each slice, were examined. Both parotid glands were delineated by a radiation oncologist. We used the Pinnacle treatment planning system to perform inverse planning and dose optimization. For each patient, IMRT plans with five or seven coplanar portals were created. Dose distributions were calculated, and separate dose-volume histograms (DVHs) were generated for each parotid gland, enabling each gland to be analyzed separately. IMRT treatment mode was used in a sequential method [3].

IMRT was delivered by a computer-controlled and auto-sequencing segmented or dynamic multileaf collimator of a linear accelerator (Varian Clinac 21 EX or Elekta Precise), with the aim of sparing the parotid glands (predominantly contralateral side) while treating the primary targets and lymph nodes at risk. The prescribed doses were 67.4 to 70.8 Gy (mean dose, 69.8 Gy) to the macroscopic tumor planning target volume (PTV1), 54.8 to 70.8 Gy (mean dose, 62.0 Gy) to the resected tumor bed planning target volume (PTV2), and 46.8 Gy to the subclinical disease planning target volume (PTV3), delivered at 1.8 to 2 Gy per fraction.

Based on the Radiation Therapy Oncology Group studies 0615, and 0225 [17], the planning objectives for PTVs were a minimum dose to >95% of the target, with no more than 5% of any PTV1 receiving ≥110% of the prescribed dose. The structural constraints used were a parotid gland mean dose of ≤26 Gy or V30Gy ≤50%; for the oral cavity excluding the PTV, the mean dose must be ≤40 Gy. The mean DVH values for the parotid gland were calculated for each patient. All data are based on mean DVHs obtained from Pinnacle3® using a bin-size resolution of 0.01 Gy. The dose calculation resolution was 2.5 mm for all IMRT plans.

### Salivary gland scintigraphy

All patients received salivary scintigraphy. Stimulated whole-mouth saliva was collected before RT and at various time intervals; for this analysis, the 1-year follow-up time point was used. Scintigraphy was performed after 4 h of fasting. After the patient received an intravenous injection of 10 mCi of 99mTc pertechnetate, sequential images of the left and right anterior views of the head and neck were acquired at 1 min/frame for 30 min. Major salivary gland function was represented by saliva excretion after sialogogue stimulation with acidic material. The salivary excretion factor (SEF) was determined as the maximal excretion activity per gland as a function of the maximal uptake [13].

Parotid gland function measured as the SEF by salivary scintigraphy was evaluated before RT and at 1 and 2 years after RT. All patients received scintigraphy 1 year after RT, whereas only 25 patients (25/31, 81%) were examined 2 years after RT. Scintigraphy was not performed for six patients because of tumor recurrence (n=2) or patient refusal (n=4). The excretion response was analyzed per patient and subsequently per individual gland. The primary end point was set as the salivary flow ≤45% of the pre-RT value [18], which is equivalent to grade 3^+^ xerostomia based on the dry mouth subscales of LENT-SOMA criteria (subjective: xerostomia, analytic: salivary flow), where grade 1 is 76–95% of pre-RT salivary flow; grade 2, 51–75%; grade 3, 26–50%; and grade 4, 0–25% [7,8].

### NTCP data fitting

All DVH data for each patient were transferred to MATLAB (version R2009b), and the analysis, including 95% confidence intervals, was performed with SPSS for Windows (version 17.0; SPSS, Chicago, IL) using the same dataset and selected variables. The data were fit to the Lyman-Kutcher-Burman (LKB) NTCP model [15,16]. The model quantitatively assesses the effects of both the radiation dose and the volume of the gland irradiated on the probability of radiation-induced changes in parotid gland function. Three parameters are represented in the sigmoidal dose–response curve: *n*, *m*, and TD_50_. The parameter *n* accounts for the volume effect of an organ: *n* was set to 1 in this study. The parameter *m* describes the slope of the dose–response curve, where decreasing *m* indicates increasing steepness of the slope. The TD_50_ is the dose for uniform irradiation of the whole or partial volume resulting in 50% probability of a complication. The NTCP is calculated from the equivalent uniform dose (EUD), assuming a sigmoidal (integrated normal distribution) relationship between the complication and EUD [19]:

(1) $$NTCP=\frac{1}{\sqrt{2\pi }}\int_{-\infty }^{t}\;e^{-\frac{x^{2}}{2}}dx$$

(2) $$t=\frac{EUD-TD_{50}}{m\cdot TD_{50}}$$

The EUD is defined as the uniform dose that would lead to the same level of tumor-cell killing as a non-uniform dose. Recently, the EUD concept has also been applied in normal tissues to evaluate the harm of a non-uniform dose distribution with the same result as a specific uniform dose. The formula for EUD is as follows:

(3) $$EUD={\left(\sum_{i=1}^{N}v_{i}D_{i}^{\frac{1}{n}}\right)}^{n}$$

where *N* is the number of voxels of the organ; *D*_*i*_ is the dose of the *i*-th voxel; *v*_*i*_ is the volume of the *i*-th voxel; and *n* is a parameter reflecting the biological properties of the organ related to its serial (0 <*n* << 1) or parallel structure (*n* of approximately 1). When *n*=1, the EUD is equal to the mean dose, as described previously [15,20,21]. The simplified LKB model represents the integral used in the Lyman formula as an exponential of a second-degree polynomial of dose.

### QoL evaluation

The EORTC questionnaire was chosen for this research because it is one of the most widely implemented questionnaires, with more than 10 years of research invested to develop an integrated, modular approach. Moreover, it has been used in international clinical trials, and the Taiwan Chinese version is easily completed by our patients. The traditional Chinese version of the EORTC QLQ-H&N35 questionnaire, obtained from the Quality of Life Unit, EORTC Data Center in Brussels, Belgium [22,23], was used for a prospective QoL survey. The primary endpoint (grade 3^+^ xerostomia) was defined as moderate to severe xerostomia 1 year after the completion of RT based on the QLQ-HN35 questionnaires. To ensure that xerostomia was induced primarily by the radiation treatment, patients with moderate to severe xerostomia at baseline were excluded from the analysis. All scales pertaining to the EORTC QLQ-H&N35 ranged from 0 to 100. A high score for a functional or global QoL scale represents a relatively high/healthy level of functioning or global QoL, whereas a high score for a symptom scale represents the presence of a symptom or problems [24-27]. All patients completed all questionnaires at three time points (before RT and at 3 and 12 months after RT), but only 17 patients (54.8%) completed the questionnaires at 2 years after RT. Thus, only the 1-year data were analyzed in the present study.

### QUANTEC guidelines

The Quantitative Analysis of Normal Tissue Effects in the Clinic (QUANTEC) guidelines are a recent concerted effort by the RT community to review and summarize normal tissue toxicity, which may suggest dose-volume treatment planning guidelines and likely reduce the rates of side effects. QUANTEC guidelines to limit the probability of severe xerostomia recommend that at least one parotid gland should receive a mean dose of ≤20 Gy or both parotid glands should receive a mean dose of ≤25 Gy [28,29]. We performed a validation test of these guidelines using prospectively collected QoL and salivary scintigraphic datasets.

### Statistical analyses

Spearman’s correlation was used to check the correlation between parotid gland excretion recovery at 1 year after RT and the mean parotid gland dose. To identify the patient- or treatment-related factors associated with parotid function recovery, we statistically analyzed age, tumor site, parotid mean dose, surgery, and chemotherapy using Spearman’s correlation and univariate and multivariate analysis. The mean scores and standard deviations of the QoL scales were calculated according to the EORTC QLQ scoring manual. To validate the QUANTEC constraints at the cut-off points of 20 Gy and 25 Gy, the negative predictive value (NPV) was checked for the rate of correctly predicting the lack of xerostomia. Positive predictive value (PPV) is the proportion of patients with xerostomia who are correctly diagnosed. The equations used for the PPV and NPV are listed in equations 4 and 5. Pearson’s chi-squared test was used to test goodness of fit and associations for both the SEF and QoL data. Overall performance was measured by Nagelkerke’s R^2^, which quantifies the amount of variation explained by the model. Model performance was also evaluated using measures for discriminative ability, including the area under the receiver operating characteristic curve (AUC). The Hosmer–Lemeshow test was used for calibration processing to test the goodness of fit for the hypothesis that the model and observed outcomes were in agreement [2]. Values of *p* < 0.05 indicated statistical significance. All analyses were performed using SPSS 17.0.

(4) $$PPV=\frac{sensitivity\times prevalence}{sensitivity\times prevalence+\left(1-specificity\right)\times \left(1-prevalence\right)}$$

(5) $$NPV=\frac{specificity\times \left(1-prevalence\right)}{\left(1-sensitivity\right)\times prevalence+specificity\times \left(1-prevalence\right)}$$

## Results

The demographic and tumor characteristics of the study population are listed in Table 1. After a median follow-up of 46.8 months (range, 34.9–62.3 months), 30 (97%) of the 31 patients were still alive. One patient had died of other disease (lung cancer). Among the 30 surviving patients, 25 were still disease free, and five patients had distant metastasis. All scintigraphic examinations and QoL assessments were performed during disease-free periods. Dosimetry analysis showed that the ipsilateral parotid gland received a dose ranging from 26.9 to 74.8 Gy (mean, 51.7 Gy), and the contralateral lobe received 7.6 to 57.6 Gy (mean, 36.7 Gy).

**Table 1:** **Patients and tumor characteristics**

**Characteristic**	**Value-*n*(%)**
**Age (y)**	
Mean	53
Range	28-78
**Gender (n)**	
Female	1 (3.2)
Male	30 (96.8)
**Tumor site**	
NPC	11 (35.5)
Oral cavity	14 (45.2)
Oropharynx	4 (12.9)
Larynx	1 (3.2)
Parotid	1 (3.2)
**Stage (TNM staging system)**	
T1	3 (9.7)
T2	12 (38.7)
T3	6 (19.4)
T4	7 (22.6)
Not applicable/Recurrent	3 (9.6)
N0	16 (51.7)
N1	5 (16.1)
N2	7 (22.6)
N3	0 (0.0)
Not applicable/Recurrent	3 (9.6)
**Dose, Gy/# fractions**	
	14 (45.2) 69.2/38
	1 (3.2) 54.8/30
	9 (29.1) 59.4/33
	4 (12.9) 57.6/32
	1 (3.2) 68.4/38
	1 (3.2) 70.8/35
	1 (3.2) 52.2/29
**Parotid gland mean dose**	
Ipsilateral, mean (range)	51.7 (26.9-74.8) Gy
Contralateral, mean (range)	36.7 (7.6-57.6) Gy
**Surgery before RT**	
Yes	16 (51.6)
No	15 (48.4)
**Chemotherapy**	
Yes	19 (61.3)
No	12 (38.7)
SEF recovery*	
Grade 3^+^ xerostomia	10 (16.1)
No grade 3^+^ xerostomia	52 (83.9)
QoL measurement*	
Grade 3^+^ xerostomia	6 (19.4)
No grade 3^+^ xerostomia	25 (80.6)

*SEF recovery and QoL measurement was at 1-year after RT. Grade 3^+^: ≧grade 3.

*Abbreviation: RT* radiotherapy, *SEF* salivary excretion factorm, *QoL* quality of life.

The SEF values before RT showed a normal distribution (mean, 48.1% ± 18.2%). Parotid gland output varied considerably, from 19.2 to 72.8%. The relationships between parotid gland excretion recovery at 1 year and patient age, tumor site, parotid gland mean dose, surgery, and chemotherapy were analyzed (Table 2). Only parotid gland mean dose was significantly correlated with recovery of parotid gland function at 1 year (r=−0.807; r^2^=0.651; *p* < 0.001).

**Table 2:** Factors analysis with parotid gland function

Factors*	**Spearman’s correlation**	**β-value**	**95% confidence interval**	**Univariate analysis (*p*-value)**	**Multivariate analysis (*p*-value)**
Parotid mean dose	−0.807	−0.759	−0.25-−0.15	< 0.001	< 0.001
Age (≥ 50 *vs.* <50)	0.146	0.141	−3.28-24.92	0.26	0.13
Tumor site (oral cavity *vs.* non-oral cavity)	−0.116	−0.101	−40.55-25.12	0.37	0.64
Surgery (yes *vs.* no)	−0.052	−0.092	−40.27-26.17	0.69	0.67
Chemotherapy (yes *vs.* no)	0.017	0.166	−1.37-27.24	0.90	0.08

*Factors associated with recovery of parotid gland function at 1 year after radiotherapy.

**Figure 1:**
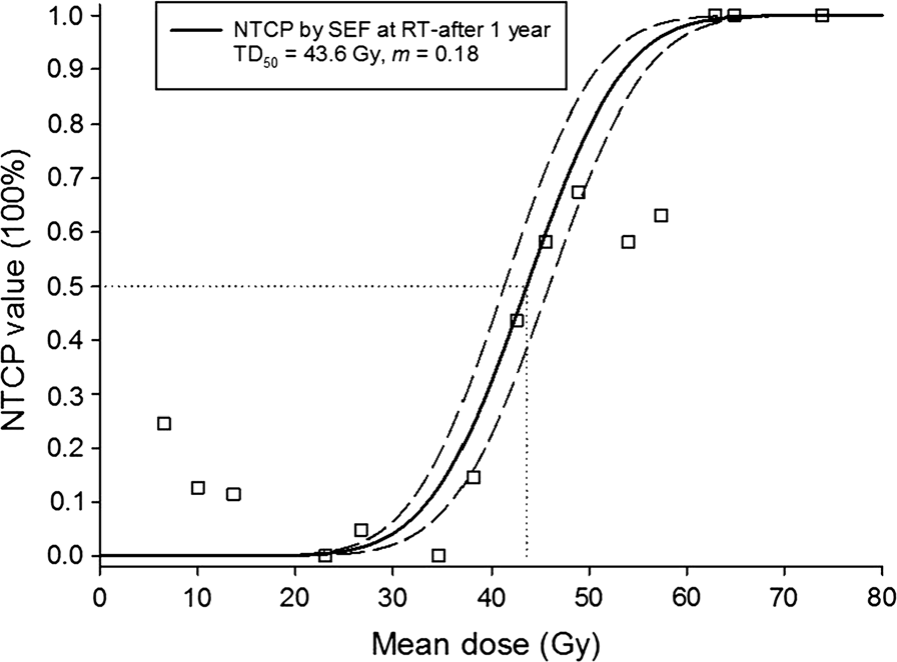
**The observed salivary excretion factor (SEF) data and the fitted dose–response curve for the normal tissue complication probability of the incidence of grade 3**^**+**^**xerostomia (salivary flow ≤45% relative to pre-RT) at 1 year after radiotherapy as a function of the mean dose to the spared parotid gland.** Dashed lines show 95% confidence intervals for the model fit to the SEF data (solid line). The squares represented the group patient mean doses in bins 4-Gy width. (All individual dose data points were used in the NTCP fitting).

**Figure 2:**
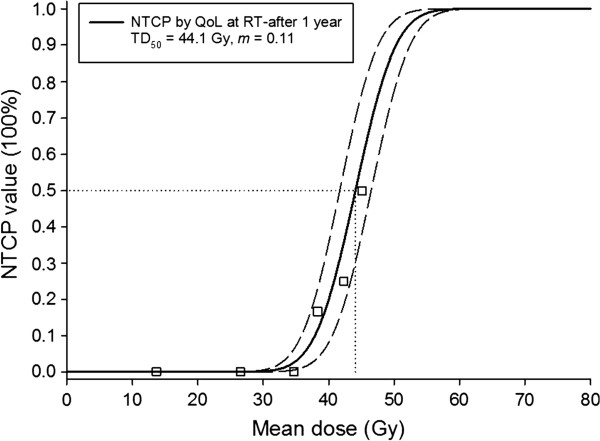
**The observed quality of life (QoL) data and the fitted dose–response curve for the normal tissue complication probability of the incidence of grade 3**^**+**^**xerostomia.** (The endpoint was defined as moderate to severe xerostomia 1 year after the completion of RT on the QLQ-HN35 questionnaires). Dashed lines show 95% confidence intervals for the model fit to the QoL data (solid line). The squares represented the average probability for groups of patients in bins 4-Gy width. (All individual data points were used in the NTCP fitting).

**Figure 3:**
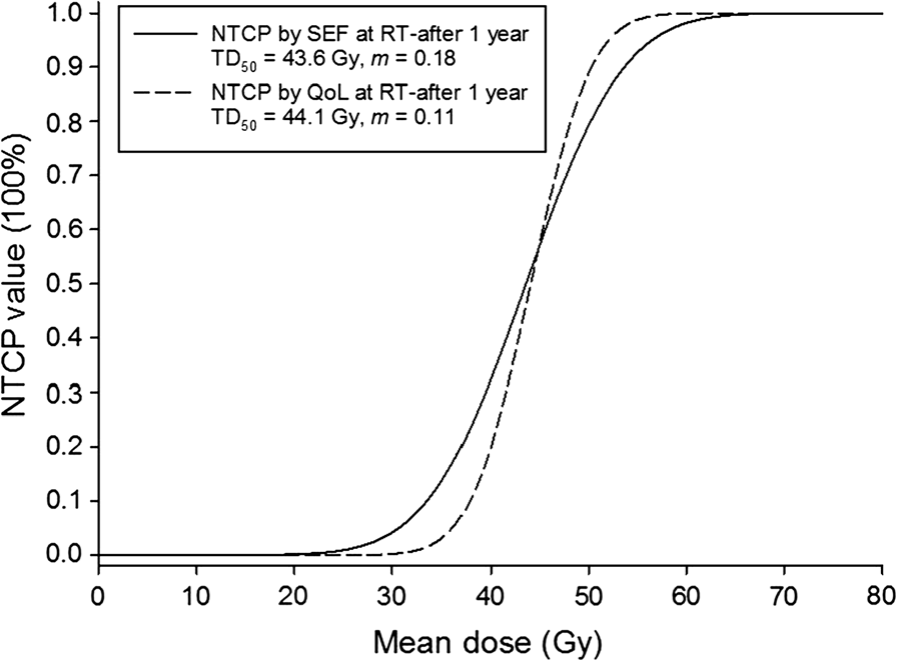
Comparison of the curves for the normal tissue complication probability (NTCP) at 1 year after radiotherapy based on the salivary excretion factor and quality of life datasets.

Figure 1 shows the observed SEF data and the fitted dose–response curves (LKB NTCP model) for the incidence of xerostomia at 1 year after completion of RT. The local fitted parameters are TD_50_=43.6 Gy (CI: 41.3-45.9 Gy) and *m*=0.18 (CI: 0.17-0.19). Dashed lines show the 95% confidence interval for the model fit to the SEF dataset (solid line). The incidences of grade 3^+^ xerostomia at 1 year were ~1% and ~2% for the recommended cut-off points of 20 and 25 Gy, respectively. Figure 2 presents the observed QoL data and fitted dose–response curves for the incidence of xerostomia at 1 year after RT. The fitted parameters are TD_50_=44.1 Gy (CI: 41.7-46.5 Gy) and *m*=0.11 (CI: 0.10-0.12). The curves for the two datasets are compared in Figure 3.

**Figure 4:**
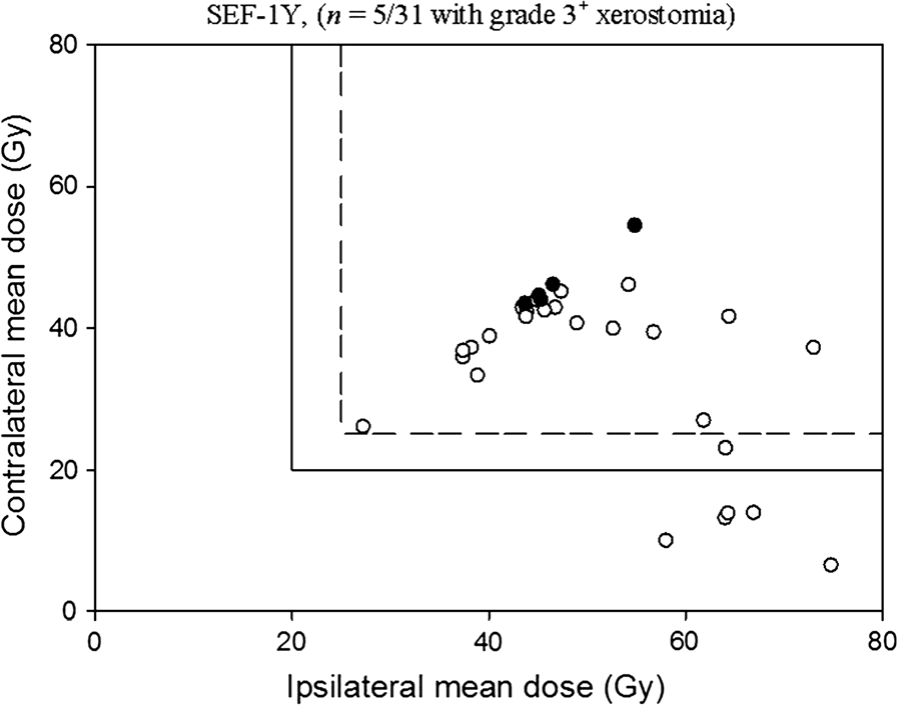
**Summary of the 25/20-Gy guidelines for radiotherapy in head and neck cancer patients applied to the salivary excretion factor (SEF) data at 1 year after radiotherapy.** The rate of xerostomia for plans meeting the QUANTEC guideline was zero, resulting in a NPV of 100% under the cut-off point. *black circle*: Grade 3^+^ xerostomia; *white circle*: No grade 3^+^ xerostomia.

**Figure 5:**
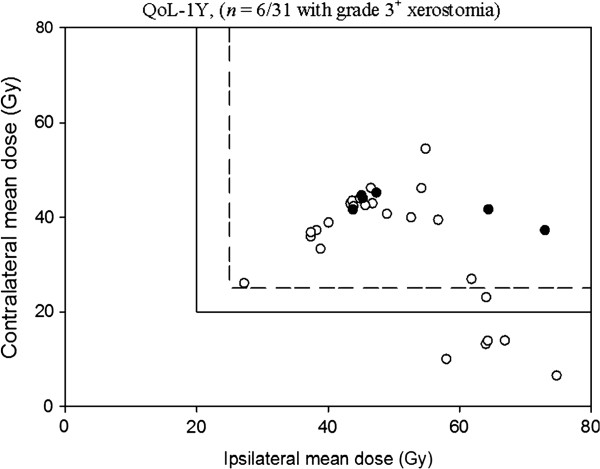
**Summary of the 25/20-Gy guidelines for radiotherapy in head and neck cancer patients applied to the quality of life (QoL) data at 1 year after radiotherapy.** The rate of xerostomia for plans meeting the QUANTEC guideline was zero, resulting in a NPV of 100% under the cut-off point. *black circle*: Grade 3^+^ xerostomia; *white circle*: No grade 3^+^ xerostomia.

The positive and negative predictive values are listed on Table 3, the cut-off points were set for the QUANTEC guidelines and our developed NTCP models. The 20- and 25-Gy QUANTEC guidelines are also applied to the SEF and QoL data in Figures 4 and 5, respectively. The incidences of xerostomia for treatment plans meeting the QUANTEC guidelines occur precisely when the spared parotid mean dose is less than the 20- or 25-Gy cut-off values, giving a NPV of 100% with each dataset. As seen, the rate of xerostomia for plans meeting our developed NTCP models are low, for 43.6 Gy cut-off point, resulting in NPV's of 92% for the SEF data and 95% for the QoL data, and for 44.1 Gy cut-off point, resulting in NPV's of 88.9% for the SEF data and 95.5% for the QoL data, respectively.

**Table 3:** Predictive values for the QUANTEC guidelines and the developed NTCP models

**Cut-off point (Gy)**	**SEF**		**QoL**	
	**PPV (%)**	**NPV (%)**	**PPV (%)**	**NPV (%)**
20_-QUANTEC_	19.2	100	23.1	100
25_-QUANTEC_	20.0	100	24.0	100
43.6_-SEF-NTCP_	50.0	92.0	45.5	95.0
44.1_-QoL-NTCP_	50.0	88.9	55.6	95.5

*Abbreviation: SEF* salivary excretion factor, *QoL* quality of life, *PPV* positive predictive value, *NPV* negative predictive value, *NTCP* normal tissue complication probability, *QUANTEC* Quantitative Analyses of Normal Tissue Effects in the Clinic.

Pearson’s chi-squared test demonstrated that the SEF and QoL data gave similar results (p = 0.241). Based on Nagelkerke’s R^2^, the overall NTCP model performance was similar between the patient-rated xerostomia-related QoL and the measured SEF values (Table 4). Furthermore, the discrimination based on the AUC was almost equal between the two datasets, and the Hosmer–Lemeshow test showed no significant disagreement between the results determined from each.

**Table 4:** Model performance and internal validation for the normal tissue complication probability model

**Performance measure**	**SEF**	**QoL**
Nagelkerke R^2^	0.12	0.11
AUC	0.75	0.75
Hosmer–Lemeshow test	χ^2^ = 10.2 (*p* = 0.24)	χ^2^ = 7.76 (*p* = 0.46)

*Abbreviation: AUC* area under the receiver operating characteristic curve; *SEF* salivary excretion factor, *QoL* quality of life.

## Discussion

Parotid gland excretion recovery at 1 year and mean parotid gland dose were strongly correlated, based on Spearman’s correlation, and mean parotid gland dose was the only significant predictive factor for xerostomia. This finding differs from the report by Beetz et al. [30,31], which proposed that multiple factors are likely to have separate impacts on xerostomia, although there may be no racial differences in the parotid gland response to irradiation.

Whole-mouth salivary function has been shown to be related to QoL determined by questionnaires [9]. With LKB NTCP modeling in the present study, the TD_50_ for xerostomia 1-year after RT was 43.6 Gy in the SEF analysis and 44.1 Gy in the QoL analysis. Although these values are higher than that reported by Moiseenko et al. (32.4 Gy) [28], they are similar to the TD_50_ reported by Dijkema et al. (39.9 Gy), who analyzed the combined and updated results from two institutions [32]. Deasy et al. suggested that the wide range of reported TD_50_ values (28.4 to 52 Gy) may result from differences in dose distribution, salivary measurement methods, segmentation, intragland sensitivity, and/or patient geographical location [29].

Xerostomia-specific questionnaires are reliable and valid for measuring patient-reported xerostomia [10]. In the present study, QoL data were shown to be as valid as SEF values for NTCP parameter modeling. Based on Pearson’s chi-squared test, SEF and QoL data gave similar results. Furthermore, both the Nagelkerke’s R^2^, which describes overall performance, and the AUC demonstrated that both datasets produced similar results, and the Hosmer–Lemeshow test showed no significant disagreement between the results determined from the SEF and QoL data. No significant difference was noted regarding dose distributions to the parotid glands.

For the IMRT planning goal, the mean dose to each parotid gland should be as low as possible while providing the desired clinical target volume coverage [33]. In our analysis, the incidence of grade 3^+^ xerostomia at 1 year was only ~1% or ~2% for the QUANTEC-recommended cut-off points of 20 Gy or 25 Gy, respectively. Hence, the severe xerostomia would usually be avoided when at least one parotid gland is spared to a mean dose ≤20 Gy or when both glands have been spared to a mean dose ≤25 Gy [29]. A lower parotid mean dose also results in better QoL for patients [34].

Potential limitations of the present study include the low number of patients with xerostomia toxicity. Although the SEF values before RT were normally distributed, confirmation in a larger sample is needed to validate the NTCP model. Grade selection for the endpoint is another potential limitation in the present study. Choosing a lower grade of xerostomia may provide more valuable dose constraints for preventing complications, as even grade 2 xerostomia significantly diminishes QoL for patients [34]. We used a previously definition for moderate to severe xerostomia based on the QLQ-HN35 questionnaire [2,30,31]. However, to our knowledge, no direct evidence exists to clarify this definition or to determine whether it is similar to the grade 3^+^ xerostomia definition by the subscales of LENT-SOMA criteria. Here, we showed the similar NTCP mapping results between moderate-to-severe xerostomia and LENT-SOMA subscales grade 3^+^ xerostomia. The practical implications of our results are validation of the use of a QoL form (EORTC QLQ-H&N35) as a surrogate for whole mouth salivary function, and also an important validation of previously proposed QAUNTEC treatment planning constraints to avoid xerostomia. Therefore, follow the QUANTEC guidelines have benefits to result in generic QoL improvement. Preserving more gland function should be pursued as a planning goal when consistent with adequate target dose coverage. Further researches will investigate as to whether new radiation techniques or different study cohorts (combining multiple institutional or cooperative group data sets) could be further validated this finding.

## Conclusions

Our study shows the agreement between the NTCP parameter modeling based on SEF and QoL data and the QUANTEC guidelines, thus validating the QUANTEC cut-off values of 20 and 25 Gy. Based on these results, we believe that the QUANTEC 25/20-Gy spared-gland mean-dose guidelines are clinically useful for avoiding xerostomia in the HN cohort.

## Competing interests

Part of this study was presented on the International Conference on Intelligent Informatics in Biology and Medicine (ICIIBM 2012).

## Authors’ contributions

TFL: original idea, study design, and writing of manuscript. PJC, HYW and PSC: statistical analysis. HCH and WCC: data collection and technical supports. All authors read and approved the final manuscript.

## Pre-publication history

The pre-publication history for this paper can be accessed here:

http://www.biomedcentral.com/1471-2407/12/567/prepub
